# Supplementary material for: The Adhesion GPCR VLGR1/ADGRV1 Regulates the Ca2+ Homeostasis at Mitochondria-Associated ER Membranes
Source: Cells. 2022 Sep 7;11(18):2790. doi: 10.3390/cells11182790 (PMC9496679; doi:10.3390/cells11182790)
Supplement: Supplementary file 1 [file cells-11-02790-s001.zip › cells-1862176-Figures S1¿CS5.pdf]

Supplementary Figures and Legends

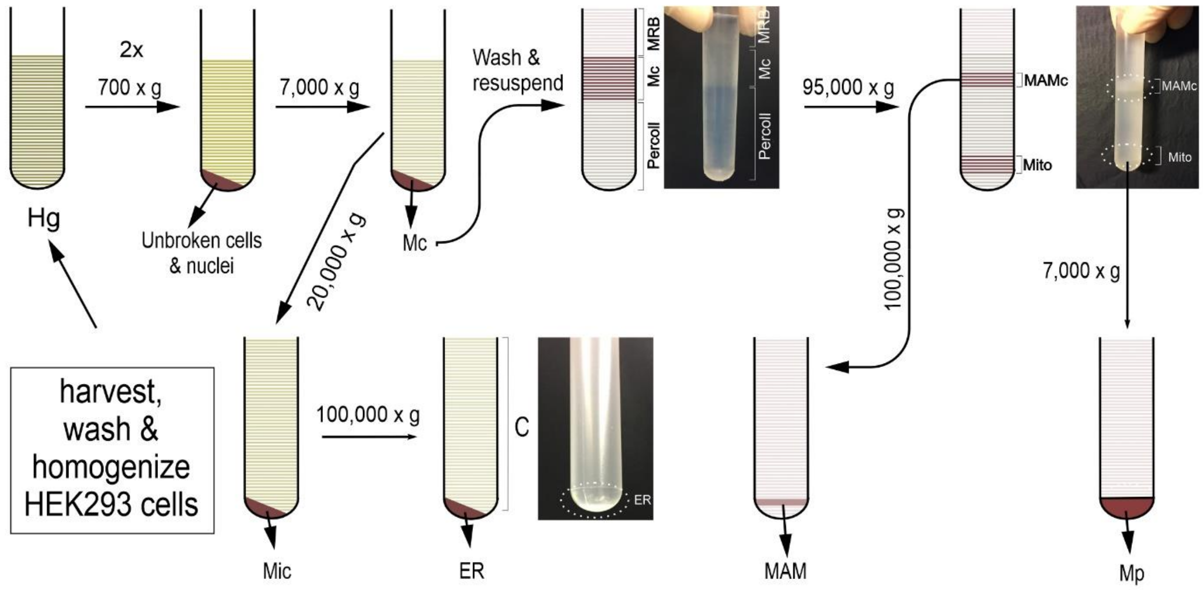

Figure S1: Step-by-step workflow of MAM isolations from HEK293T cells by differential centrifugation. Details of the protocol are described in the text. Abbreviations: Hg, cell homogenate; Mc, crude mitochondria fraction; Mic, microsomes; C, cytosol fraction; ER, endoplasmic reticulum fraction; MAM, mitochondria-associated ER membrane (MAM) fraction; MAMc, crude MAM band; Mito, "mitochondria" band; Mp, pure mitochondria fraction**.**

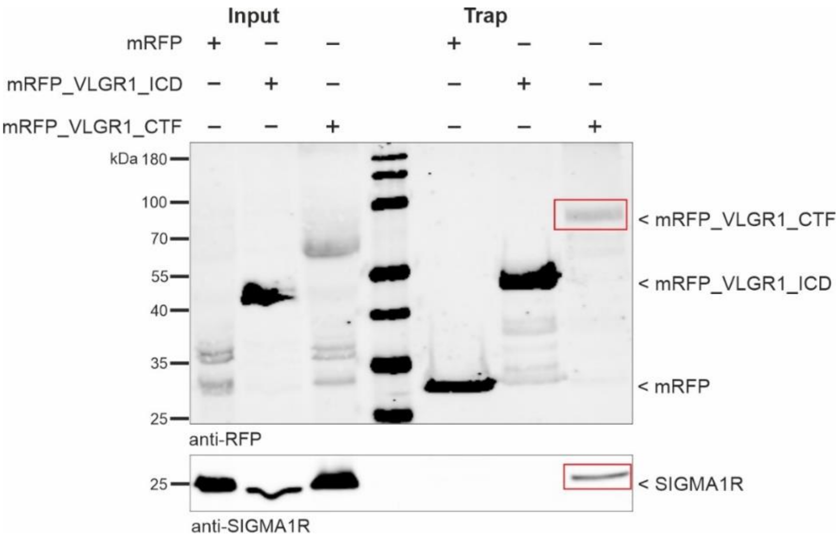

Figure S2: S1R (SIGMA1R) interacts with VLGR1_CTF, but not with VLGR1_ICD. Western Blot analysis of RFP-Trap® from lysates of HEK293T cells expressing VLGR1_CTF-mRFP, VLGR1_ICD-mRFP or mRFP. Intrinsic S1R (SIGMA1R) was precipitated by VLGR1_CTF-mRFP, but not by VLGR1_ICD-mRFP or mRFP.

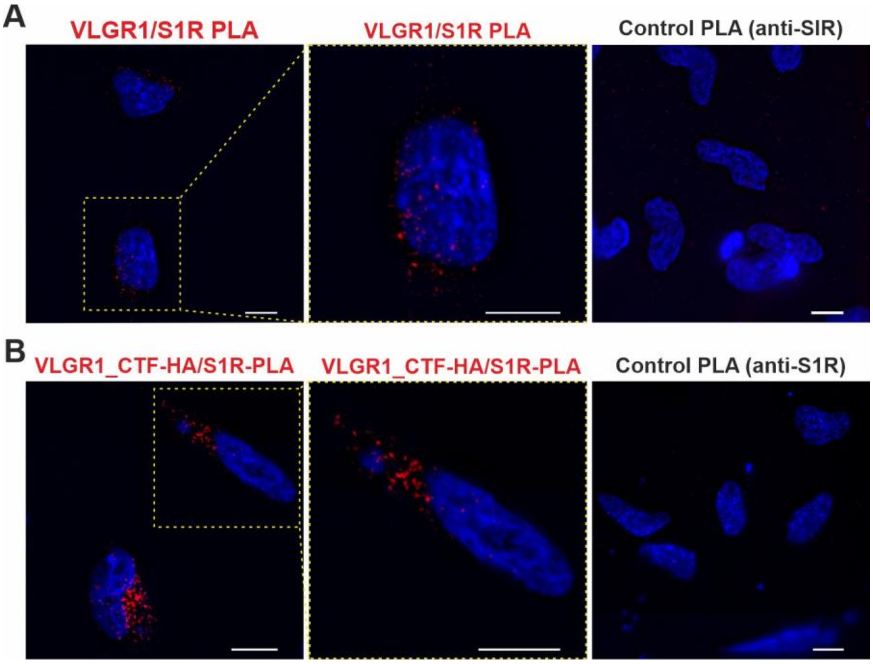

Figure S3: Proximity ligation assay (PLA) with VLGR1 with S1R in HeLa cells. (A) PLA assay for intrinsic VLGR1 and S1R. Red PLA signals represent colocalization of both proteins. Both, anti-S1R or anti-VLGR1 (not shown) only were probed with both PLA (rabbit, rb and mouse, ms) secondary antibodies including PLUS and MINUS complementary sequences and used as negative control. **(B)** PLA assay for VLGR1_CTF fragment and SIGMA1R. Colocalization is indicated by red PLA signals. Anti-S1R and anti-VLGR1 (not shown) only with PLA (rabbit, rb and mouse, ms) secondary antibodies including PLUS and MINUS complementary sequences were used as negative control, no signal was observed in the control. All images of the respective replicates were acquired using the same light exposure and intensity settings. Nuclei were stained with DAPI (blue). Bars: 5 µm and 10 µm in controls.

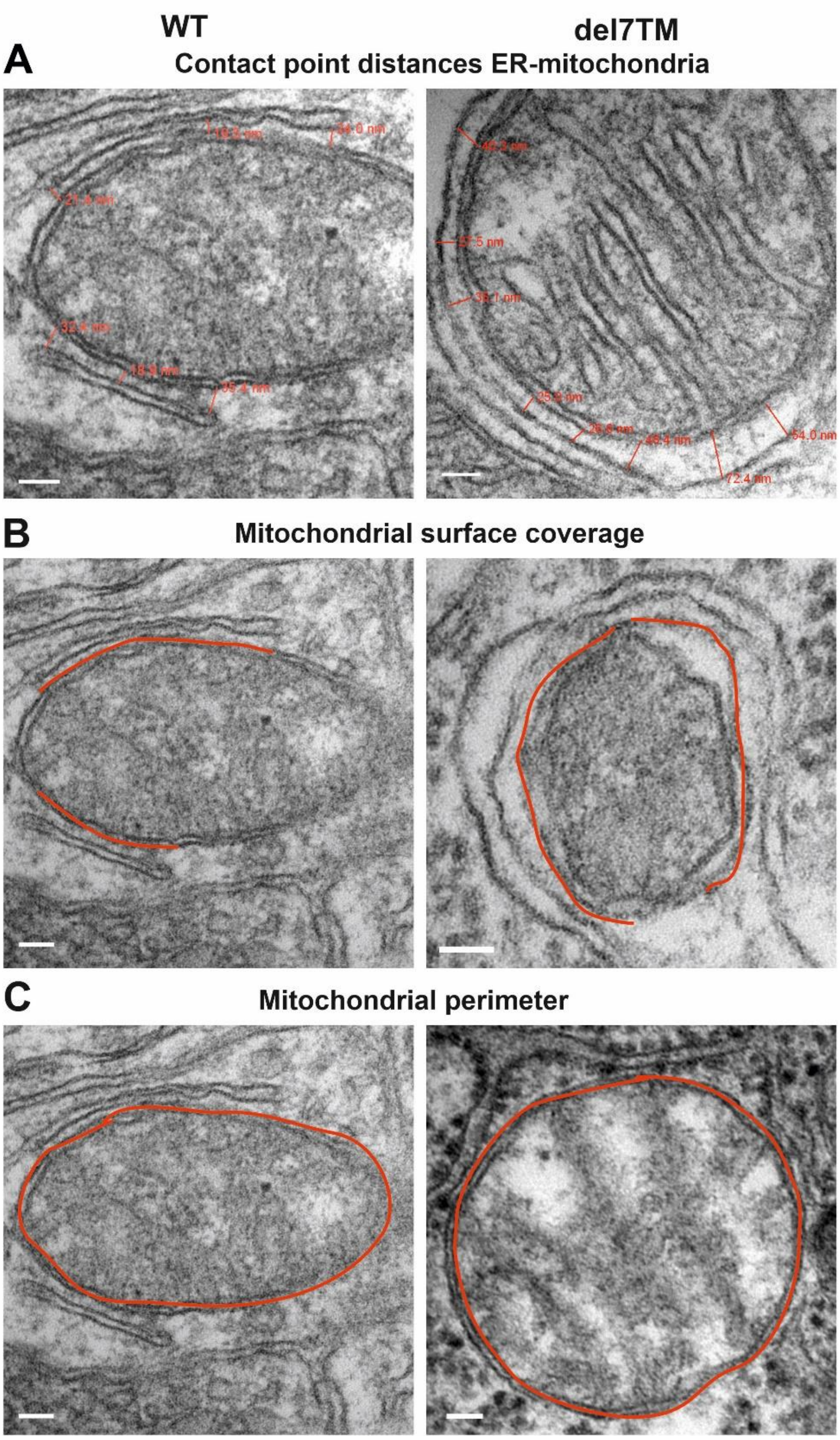

Figure S4: Representative TEM images of morphometric analyses of the ER-mitochondria interface, the mitochondrial surface coverage and mitochondrial parameter in cerebellar neurons of WT and del7TM mice. **(A)** Representative TEM images of the measurement of contact point distances at the ER-mitochondrial interface. **(B)** Representative TEM images of measurements of the mitochondrial surface by ER indicated by red lines. **(C)** Representative TEM images of the mitochondrial perimeter indicated in red. Bars: 50 nm.

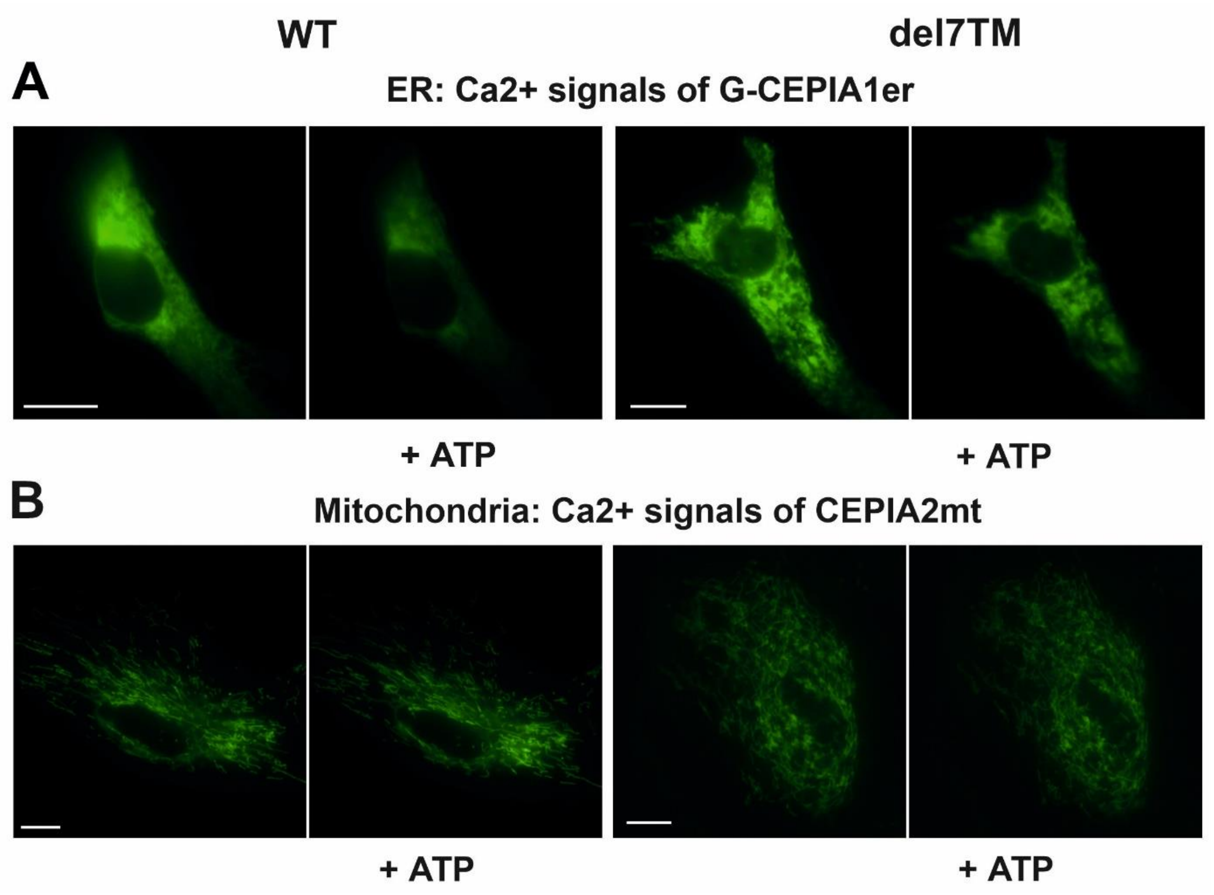

Figure S5: Representative fluorescence images of Ca^{2+} signals of G-CEPIA1er in the ER and of CEPIA2mt in mitochondria before and after stimulation via ATP in murine primary astrocytes in WT and *Vlgr1*del7TM astrocytes. (A) Ca^{2+} signals of G-CEPIA1er in the ER **(B)** Ca^{2+} signals of CEPIA2mt in mitochondria. Bars: 10 µm.
